# Determining the *Bordetella* LPS structural features that influence TLR4 downstream signaling

**DOI:** 10.3389/fmicb.2025.1540534

**Published:** 2025-02-25

**Authors:** Kiruthika Manivannan, Yasmine Fathy Mohamed, Rachel C. Fernandez

**Affiliations:** ^1^Department of Microbiology and Immunology, Life Sciences Institute, The University of British Columbia, Vancouver, BC, Canada; ^2^Department of Microbiology and Immunology, Faculty of Pharmacy, Alexandria University, Alexandria, Egypt

**Keywords:** *Bordetella*, LPS, TLR4, MyD88, NFκB, TRIF, IRF3

## Abstract

Upon recognizing bacterial lipopolysaccharide (LPS), human TLR4 initiates two distinct signaling pathways: the MyD88 pathway from the cell surface or the TRIF pathway following endocytosis. While the first is associated with strong pro-inflammatory responses, the latter is linked to dendritic cell maturation and T cell priming. Changes in LPS structure can influence the activation of either or both pathways. This study investigates the influence of specific structural features of *Bordetella* LPS on these pathways: the O antigen, the number of acyl chains in lipid A and the glucosamine modification of the phosphates of the lipid A diglucosamine backbone. Systematically engineered *Bordetella* LPS differing in one or more of these features were studied by quantifying NFκB and IRF3 activation—indicators of MyD88 and TRIF pathway activation, respectively. The findings reveal that the glucosamine modification of lipid A plays a dominant role in TLR4-mediated signaling, overriding the influence of the O antigen and lipid A acylation. The absence of glucosamine modification significantly reduced the activation of both MyD88 and TRIF pathways, underscoring its importance in promoting TLR4 dimerization. Furthermore, under-acylation of LPS (with 4 or 5 acyl chains) partially reduced NFκB activation, while completely abrogating TRIF pathway activation. In contrast, hexa-and hepta-acylated LPS equally and robustly activated both pathways. Lastly, the *Bordetella* O antigen selectively biased signaling towards the TRIF pathway without affecting the MyD88 pathway. This study provides valuable insights into how specific LPS structural modifications can be leveraged to tailor TLR4-mediated signaling.

## 1 Introduction

Lipopolysaccharide (LPS) is the predominant component of the outer membrane of most Gram-negative bacteria. Its structure is typically composed of lipid A, core sugars and, in some bacteria, O antigen polysaccharide. The lipid A, in turn, consists of 4 to 7 acyl chains attached to a diglucosamine backbone. Together, this glycolipid entity is highly immunodominant and a key bacterial virulence factor.

The human innate immune system mainly detects LPS using the pattern recognition receptor complex, Toll-like receptor-4/Myeloid differentiation factor-2 (TLR4/MD-2) (Lu et al., 2008; Ciesielska et al., 2021). Its cofactors, LPS binding protein and CD14, relay LPS to TLR4/MD-2 from the bacterial surface or from solution. When lipid A of LPS binds to the pocket of MD-2, it triggers the dimerization of two TLR4/MD-2—LPS complexes and initiates one of two signaling cascades. The dimer can signal from the cell surface using the MyD88-dependant pathway to activate the transcription factors, NFκB and/or AP-1, and thereby induce a strong pro-inflammatory response. Additionally, the dimer can also be endocytosed where it interacts with adaptors, TRAM and TRIF, to induce IRF3-mediated type-I interferons, which thereby promote dendritic cell (DC) maturation and differentiation and consequently impact T cell priming. TRIF-mediated signaling also leads to the late-phase activation of NFκB and AP-1. Alternative to LPS recognition by TLR4/MD-2, endocytosed or cytosolic LPS can also be detected by caspases-4/11 to activate inflammasomes (Zamyatina and Heine, 2020).

Consequently, bacteria have evolved to evade or temper the immune response generated by altering their LPS structure. Modification to the LPS biosynthesis pathway itself can alter the lipid A backbone or acyl chain characteristics as well as change the properties of or even completely replace the O antigen. Bacteria also have mechanisms in place to alter their LPS post-synthesis largely under the regulation of two-component systems. Examples include the addition, removal or hydroxylation of acyl chains, altering the charge of lipid A or addition of groups to the core oligosaccharide (Bertani and Ruiz, 2018; Simpson and Trent, 2019). All these factors, put together, alter its interaction with TLR4/MD-2 and subsequently, the degrees to which the downstream pathways are activated.

The traditional hexa-acylated *E. coli* LPS is an agonist of TLR4, strongly activating both MyD88 and TRIF pathways. In contrast, under-acylated LPS usually act as antagonists as reducing the number of acyl chains alters their fit in the MD-2 pocket (Park et al., 2009). For example, penta-acylated *R. sphaeroides* LPS does not trigger TLR4-mediated signaling and, in fact, competitively inhibits the binding of other agonistic LPS (Qureshi et al., 1991; Kirikae et al., 1994; Stevens et al., 2013; Anwar et al., 2015). In the middle of these extremes lies monophosphoryl lipid A (MPLA), an FDA-approved adjuvant that is a chemically detoxified LPS isolated from *Salmonella minnesota* Re595. MPLA has been shown to greatly reduce the MyD88-dependent pro-inflammatory response while preserving signaling via the TRIF pathway (Mata-Haro et al., 2007).

LPS also forms a part of the outer membrane of *Bordetella* spp., a group of small, Gram-negative, coccobacilli that cause a highly contagious respiratory disease in a wide range of hosts, from humans to various mammals including sheep, pigs and mice, as a well as poultry and wild birds (Rivera et al., 2020). The *Bordetella* spp. have evolved, potentially due to the different selection pressures faced in the different hosts, to express a wide variety in LPS structures. *B. pertussis* is a penta-acylated member devoid of an O antigen in the otherwise predominantly hexa-acylated, O antigen-expressing *Bordetella* family. Some species (*B. pertussis*, *B. bronchiseptica*, *B. parapertussis*, and *B. avium*) modify their backbone phosphates with charged glucosamine (GlcN) moieties (Marr et al., 2008; Novikov et al., 2014; Novikov et al., 2019), while some (*B. pertussis*, *B. bronchiseptica*, *B. avium*, and *B. hinzii*) decorate their core with a distal trisaccharide (Preston et al., 2006; El Hamidi et al., 2009; Novikov et al., 2019). Additional diversity in the acyl chain length, distal trisaccharide and the O antigen composition is brought about by genetic diversity in the genes and loci involved in their biosynthesis and ligation (Novikov et al., 2019). Much remains unknown as to how these structural differences in *Bordetella* LPS influence their recognition by the host TLR4/MD-2 and the subsequent downstream signaling.

Of all *Bordetella* species, human respiratory illness (whooping cough) is mainly caused by *B. pertussis* and *B. parapertussis*, with *B. pertussis* causing the more severe disease. The number of cases of *B. pertussis* infection is resurging in many parts of the world, including USA, Canada, and Europe in 2024 with case numbers surpassing those in 2019 after a brief respite due to COVID-19 pandemic-related restrictions.^1^^,^^2^^,^^3^ In parallel, *B. parapertussis* is also reemerging, with a significant increase in its detection rates in PCR-tested samples in 2023 (Noble et al., 2024). Hence, these strains warrant further investigation, and this study focused on the recognition of *B. pertussis* and *B. parapertussis* LPS variants by human TLR4.

Despite infecting the same host, *B. pertussis* and *B. parapertussis* have evolved to exhibit significant differences in their LPS structure. *B. parapertussis* encodes a homopolymeric O antigen while *B. pertussis* does not (Di Fabio et al., 1992; Preston and Maskell, 2001). Studies using *E. coli* LPS implicate the O antigen in biasing TLR4-mediated signaling towards the TRIF pathway by interacting with the cofactor, CD14 (Gangloff et al., 2005; Jiang et al., 2005; Zanoni et al., 2012). Fedele et al., 2008 showed that purified *B. parapertussis* LPS (with an O antigen) induced significantly lower monocyte-derived DC (MDDC) maturation in the absence of CD14, while MDDC maturation triggered by purified *B. pertussis* LPS (without an O antigen) was not affected by the absence of CD14. Thus, the O antigen was implicated in playing a role in CD14-mediated LPS signaling (Fedele et al., 2008). Our study utilized systematically engineered *Bordetella* LPS with and without the O antigen to delineate its influence on the activation of TLR4-mediated signaling pathways: the MyD88 pathway and, particularly, the CD14-dependent TRIF pathway.

Another feature differentiating the strains is the number of acyl chains. *B. pertussis* is penta-acylated, while *B. parapertussis* is hexa-acylated. Under-acylation in Gram-negative bacteria has been associated with reduced TLR4 signaling (Meng et al., 2010; Herath et al., 2013; Anwar et al., 2015). Similarly, in *Bordetella* spp., hexa-acylated species (either *B. parapertussis* or *B. pertussis* modified to encode a hexa-acylated structure) were shown to activate NFκB mediated responses to a greater degree than penta-acylated *B. pertussis* (Geurtsen et al., 2009; Fathy Mohamed and Fernandez, 2024). This study expanded on these findings, investigating the activation of both TLR4-mediated signaling pathways by *Bordetella* LPS that expressed a wider variation in acyl chain numbers (from 4 to 7 acyl chains).

Lastly, both *B. pertussis* and *B. parapertussis* decorate the phosphates of the diglucosamine backbone with GlcN moieties (Marr et al., 2008). This modification has been shown to promote both the MyD88 (Geurtsen et al., 2009; Marr et al., 2010a; Marr et al., 2010b) and the TRIF pathway (Marr et al., 2010a) by influencing TLR4 dimerization (Maeshima et al., 2015). This study examined the influence of the GlcN moiety on TLR4-mediated signaling in combination with other structural modifications of *Bordetella* LPS, i.e., the presence or absence of the O antigen and the alteration in the number of acyl chains.

Even minor differences in LPS structure alter the LPS’ properties and its interaction with the host TLR4/MD-2 (Miller et al., 2005; Maeshima and Fernandez, 2013). Thus, the extent to which the MyD88 and the TRIF pathways are activated is affected, which consequently influences downstream adaptive immune responses (Fitzgerald and Kagan, 2020; Duan et al., 2022). In this study, we found that the GlcN modification of *Bordetella* LPS increases the activation of both MyD88 and TRIF pathways, irrespective of alterations to the other structural features studied. Additionally, while an extra acyl chain in *Bordetella* LPS did not alter TLR4 signaling, under-acylation partially reduced NFkB responses and failed to activate the TRIF pathway. Finally, the *Bordetella* O antigen biased signaling towards the TRIF pathway without affecting the MyD88 pathway activation. Altogether, this study highlights the nuanced interplay of these structural features in activating TLR4-mediated signaling pathways.

## 2 Materials and methods

### 2.1 Bacterial strains, plasmids, and growth conditions

All strains and plasmids used in this study are listed in Table 1. Bacteria were grown as described before (Ifill et al., 2021). As needed, media were supplemented with nalidixic acid (Nal; 30 μg/mL), gentamicin (Gm; 15 μg/mL), kanamycin (Kan; 75 μg/mL), diaminopimelic acid (DAP; 250 μg/mL) and/or anhydrous tetracycline (aTC; 12.5 ng/mL).

**Table 1:** List of bacterial strains and plasmids used in the study.

Strain or plasmid	Description	References/source/notes
*E. coli* strains		
DH5α	Molecular cloning strain	Invitrogen
RHO3	Conjugation strain, Δasd ΔaphA, DAP auxotroph	Lopez et al. (2009) and de Jonge et al. (2021)
*B. pertussis* strains		
BP338 (WT)	Wild type *B. pertussis* Tohama-1 strain; Nal^r^	Alison Weiss (University of Cincinnati)
BP338 Δ*lgmA-D*	BP338 with the *lgm* locus deleted; Nal^r^	Shah et al. (2013)
BP338 Δ*lgmA-D* Comp	BP338 Δ*lgmA-D* with the *lgm* locus complemented using the mini-Tn7 transposon system; Nal^r^, Kan^r^	This study
*B. parapertussis* strains		
BPP12822 WT	Wild type *B. parapertussis* strain 12822	ATCC BAA-587
BPP12822 Δ*lgmA-D*	BPP12822 with the *lgm* locus deleted	This study
BPP12822 Δ*waaL*	BPP12822 with the O antigen ligase, WaaL, deleted	This study
BPP12822 Δ*lgmA-D* Δ*waaL*	BPP12822 with the *lgm* locus and *waaL* deleted sequentially	This study
BPP12822 Δ*wbmA-E*	BPP12822 with the first five genes of the O antigen biosynthesis locus (*wbmA-E*) deleted	This study
BPP12822 Δ*lgmA-D* Δ*wbmA-E*	BPP12822 with the *lgm* locus and *wbmA-E* deleted sequentially	This study
BPP12822 Δ*pagP*	BPP12822 with the palmitoyl transferase, PagP, deleted	This study
BPP12822 Δ*lgmA-D* Δ*pagP*	BPP12822 with *lgm* locus and *pagP* deleted sequentially	This study
BPP12822 Δ*pagL*	BPP12822 with the deacylase, PagL, deleted	This study
BPP12822 Δ*pagP* Δ*pagL*	BPP12822 with *pagP* and *pagL* deleted sequentially	This study
BPP12822 Δ*lgmA-D* Comp	BPP12822 Δ*lgmA-D* with the *lgm* locus complemented using the mini-Tn7 transposon system; Kan^r^	This study
BPP12822 Δ*waaL* Comp	BPP12822 Δ*waaL* with *waaL* complemented on the aTC inducible pIG10 plasmid; Gm^r^	This study
BPP12822 Δ*lgmA-D* Δ*waaL* Comp	BPP12822 Δ*lgmA-D* Δ*waaL* with the *lgm* locus complemented using the mini-Tn7 transposon system and *waaL* on pIG10; Kan^r^, Gm^r^	This study
BPP12822 Δ*wbmA-E* Comp	BPP12822 Δ*wbmA-E* with *wbmA-E* complemented on pIG10; Gm^r^	This study
BPP12822 Δ*lgmA-D* Δ*wbmA-E* Comp	BPP12822 Δ*lgmA-D* Δ*wbmA-E* with the *lgm* locus complemented using the mini-Tn7 transposon system and *wbmA-E* on pIG10; Kan^r^, Gm^r^	This study
BPP12822 Δ*pagP* Comp	BPP12822 Δ*pagP* with *pagP* complemented on pIG10; Gm^r^	This study
BPP12822 Δ*lgmA-D* Δ*pagP* Comp	BPP12822 Δ*lgmA-D* Δ*pagP* with the *lgm* locus complemented using the mini-Tn7 transposon system and *pagP* on pIG10; Kan^r^, Gm^r^	This study
BPP12822 Δ*pagL* Comp	BPP12822 Δ*pagL* with *pagL* complemented on pIG10; Gm^r^	This study
BPP12822 Δ*pagP* Δ*pagL* Comp	BPP12822 Δ*pagP* Δ*pagL* with *pagP* and *pagL* (separated by a ribosome-binding sequence) complemented on pIG10; Gm^r^	This study
Plasmids		
pSS4894	Suicide vector containing I-SceI restriction enzyme under Ptx promoter and cognate restriction site, used for allelic exchange; Gm^r^	Chen et al. (2017)
pSS4245—Δ*lgmA-D*	pSS4245 containing ~600-bp long upstream and downstream regions of *B. pertussis lgm* locus required for markerless deletion	Shah et al. (2013)
pIG02	Derived from pSS4894 suicide vector containing I-SceI restriction enzyme and cognate restriction site, used for allelic exchange; Gm^r^	Chen et al. (2017)
pIG02—Δ*waaL*	pIG02 containing ~600-bp long upstream and downstream regions of *B. parapertussis waaL* for markerless deletion of *waaL*; Gm^r^	This study
pIG02—Δ*wbmA-E*	pIG02 containing ~600-bp long upstream and downstream regions of *B. parapertussis wbmA-E* for markerless deletion of *wbmA-E*; Gm^r^	This study
pIG02—Δ*pagP*	pIG02 containing ~600-bp long upstream and downstream regions of *B. parapertussis pagP* for markerless deletion of *pagP*; Gm^r^	This study
pIG02—Δ*pagL*	pIG02 containing ~600-bp long upstream and downstream regions of *B. parapertussis pagL* for markerless deletion of *pagL*; Gm^r^	This study
pIG10	Tetracycline inducible gene expression vector, derived from pT10 and optimized for use in *B. pertussis*. Incorporates into *B. pertussis* genome, Amp^r^ Gm^r^	Ifill and Fernandez, manuscript in preparation
pIG10—Bpp *waaL*	*B. parapertussis waaL* cloned into pIG10	This study
pIG10—Bpp *wbmA-E*	*B. parapertussis wbmA-E* cloned into pIG10	This study
pIG10—Bpp *pagP*	*B. parapertussis pagP* cloned into pIG10	This study
pIG10—Bpp *pagL*	*B. parapertussis pagL* cloned into pIG10	This study
pIG10—Bpp *pagP pagL*	*B. parapertussis pagP* and *pagL* cloned into pIG10 separated by a ribosome-binding site: 5′-GGCAAGTCTAAAGCCATAGAAGGATAC-3′	This study
pUC18T-mini-Tn7T-Km-*FRT*	Mobilizable transposition vector; Amp^r^, Kan^r^	Choi et al. (2008)
pTNS2	Tn7 transposase vector for expression of *tnsABCD*, Amp^r^	Choi et al. (2008) and Anwar et al. (2015)
pUC18-miniTn7T-*lgmA-D*	Tn7 transposon containing the *lgm* locus with ~1,000 bp upstream to include native promoters, Amp^r^, Kan^r^	Gyles Ifill

### 2.2 Markerless gene deletion in *Bordetella* species

A markerless clean deletion protocol was used to delete the genes of interest: *lgmA-D*, *waaL*, *wbmA-E*, *pagP* and *pagL* in *B. parapertussis* as described previously (Marr et al., 2010a; Shah et al., 2013; Ifill et al., 2021). Due to the sequence similarity in the *lgm* locus between *B. pertussis* and *B. parapertussis*, the same plasmid (pSS4245 Δ*lgmA-D*) used to delete the locus in *B. pertussis* (Shah et al., 2013), was used in *B. parapertussis* as well. pIG02 was used for the clean deletion of other genes by cloning ~600-bp of the upstream and downstream regions of the genes of interest [separated by an SpeI restriction enzyme (RE) site] between the KpnI and BamHI RE sites in its multiple cloning site (MCS). This construct was then transformed into *E. coli* RHO3. These allelic exchange plasmids were then conjugated into *B. parapertussis* using the di-parental mating protocol as previously published (Ifill et al., 2021) with the noted absence of nalidixic acid (*B. parapertussis* strain is not Nal^r^). In the case of double mutants, the allelic exchange protocol was repeated with the generated single gene deletion mutant and the RHO3 strain containing the pIG02 construct for the clean deletion of the second gene of interest.

### 2.3 Complementation of deleted genes

The *B. parapertussis waaL*, *wbmA-E*, *pagP* and *pagL* deletion mutants were complemented with their respective genes using pIG10, an anhydrous tetracycline (aTC) inducible expression plasmid created for *Bordetella* species by Gyles Ifill (Ifill and Fernandez, manuscript in preparation). The gene of interest was amplified and cloned into the MCS of pIG10 between the SpeI and BamHI RE sites. In the case of *B. parapertussis* Δ*pagP* Δ*pagL*, both genes were cloned into pIG10 separated by a HindIII RE site and a ribosome-binding sequence, 5′-GGCAAGTCTAAAGCCATAGAAGGATAC-3′ to ensure the expression of both genes. The construct was introduced into the respective mutants using di-parental conjugation as described above.

The *lgm* locus, along with ~1,000-bp of the upstream region (to include its native promoter), was introduced into the chromosome using the mini-Tn7 transposon delivery plasmid, pUC18T-mini-Tn7T-Km-FRT, at the attTn7 site located downstream of the highly conserved and essential *glmS* genes (Choi et al., 2005). The construct, and its transposase vector, pTNS2, were introduced into the Δ*lgmA-D* mutants using tri-parental conjugation. This method, similar to di-parental mating described above, involved mixing *E. coli* RHO3 containing the pUC18-miniTn7T-*lgmA-D* construct, *E. coli* RHO3 carrying pTNS2 and the *B. parapertussis* mutant strain in the ratio of 1:1:2. Kanamycin was used as the selection antibiotic. Successful integration of the transposon was confirmed by PCR.

### 2.4 Tricine-SDS-PAGE

*B. pertussis* and *B. parapertussis* strains were grown in liquid culture inoculated at an initial OD_600_ of 0.001. They were grown to an OD_600_ of 0.6–0.8 (up to 72 h under agitation). 1.5 mL of bacterial suspension (concentrated to an OD_600_ of 2) was digested with DNase I, RNase and proteinase K, and the resulting lysate was separated using tricine-SDS-PAGE and visualized using silver staining (Marolda et al., 2006).

### 2.5 MALDI-TOF analysis

To prepare cells for MALDI-TOF analysis, 100 mL of *B. pertussis* or *B. parapertussis* liquid culture was grown as stated above. The bacteria were harvested, and lipid A was extracted using the ammonium-isobutyrate method (El Hamidi et al., 2005) and analyzed in the Applied Biosystems MALDI-TOF spectrometer as described previously (Fathy Mohamed and Fernandez, 2024). Data was acquired and analyzed using the Data Explorer software and graphed using GraphPad Prism 10 (RRID:SCR_002798).

### 2.6 Cell lines

HEK-Blue^™^ hTLR4 cells (InvivoGen Cat# hkb-htlr4) and HEK-Blue^™^ Null2 cells (InvivoGen Cat# hkb-null2) were cultured as described previously (Shah et al., 2013). HEK-Blue^™^ hTLR4 cells are engineered from HEK293 cell line to stably express human TLR4, MD2, CD14 and an inducible secreted embryonic alkaline phosphatase (SEAP) reporter gene to measure NFκB activation. HEK-Blue^™^ Null2 is the parental cell line of HEK-Blue^™^ hTLR4 expressing the SEAP reporter alone to exclude NFκB responses induced by the activation of endogenously expressed pattern recognition receptors, including TLR3, TLR5 and RIG-1-like receptors.

THP1-Dual^™^ cells (InvivoGen Cat# thpd-nfis) were cultured in RPMI 1640 (GIBCO) containing 10% heat-inactivated (30 min at 56°C) fetal bovine serum (Sigma), 2 mM GlutaMAX (GIBCO), 25 mM HEPES (GIBCO), 100 μg/mL Normocin (InvivoGen), and Penicillin-Streptomycin (100 U/mL-100 μg/mL; GIBCO) in the presence of selection antibiotics: 100 μg/mL zeocin (InvivoGen) and 10 μg/mL blastocidin (InvivoGen). They were passaged after reaching densities of 1–2 × 10^6^ cells/mL. THP1-Dual^™^ cells are engineered from human THP-1 monocyte cell line to express two inducible reporter genes, SEAP to measure NFκB activation and Lucia luciferase to measure IRF3 activation.

THP-1 cells (ATCC) were cultured as described previously (Fathy Mohamed and Fernandez, 2024) and passaged after reaching a density of 1 × 10^6^ cells/mL.

All cell lines were incubated in a CO_2_ incubator at 37°C with 5% CO_2_.

### 2.7 Preparation of bacterial strains and controls for assessing TLR4 activation

All *Bordetella* strains, and the positive control, *E. coli* DH5α, were grown to an OD_600_ of 0.6–0.8. They were concentrated to an OD_600_ of 5 in phosphate-buffered saline (PBS) and heat-killed at 60°C for 1 h. The lack of viability was confirmed by spotting a small aliquot (2 μL) on agar plates and checking for the lack of bacterial growth after incubating the plates for up to 5 days. The heat-killed samples were stored at −20°C.

*E. coli* K12 LPS (InvivoGen) was resuspended as recommended and stored in aliquots at −20°C. When needed, an aliquot was thawed, placed in a sonicating water bath for 10 min and then used to prepare required dilutions for the respective assays.

### 2.8 HEK-Blue NFκB reporter assay

HEK-Blue^™^ hTLR4 cells and HEK-Blue^™^ Null2 cells were grown to ~70–80% confluency. Then the reporter assay was carried out as described previously (Shah et al., 2013) using the indicated dilution of heat-killed bacterial suspension as stimulants or media for negative control. The alkaline phosphatase reporter activity was quantified by measuring the absorbance after the indicated incubation period with the QUANTI-Blue reagent (Invivogen) at 650 nm in the Molecular Devices SpectraMax 190 microplate reader or the Thermo Scientific VarioSkan Flash multimode plate reader. Readings were converted as a percentage of *B. parapertussis* WT. One-way ANOVA with Tukey’s multiple comparison test was performed using GraphPad Prism 10.

### 2.9 THP-1 Dual^™^ IRF3 reporter assay

One hundred and eighty microliters of THP-1 Dual^™^ cells (~100,000 cells/well) were aliquoted per well of 96-well flat-bottomed, tissue culture-treated plates (Corning, Cat# 353072). They were differentiated into macrophages by treating them with 50 ng/mL phorbol 12-myristate 13-acetate (PMA; Sigma-Aldrich) for 48 h at 37°C in a CO_2_ incubator. The cells were then washed with fresh media twice to remove traces of PMA and rested for 72 h. On day 4, the cells were washed again, and 180 μL of fresh media was added per well. Subsequently, 20 μL of stimulant prepared in media was added per well to obtain the desired final concentration of stimulant (1:100 dilution of heat-killed bacterial suspension; endotoxin-free water for negative control; 1 μg/mL *E. coli* K12 LPS as positive control). After incubation at 37°C for 24 h, 10 μL of the supernatant was used to determine the luciferase activity using the QUANTI-Luc 4 Lucia/Gaussia reagent (InvivoGen) as per manufacturer’s flash detection protocol in the Perkin-Elmer Victor X5 Multilabel reader. Readings were converted as a percentage of *B. parapertussis* WT. Mixed-effects analysis with Tukey’s multiple comparison test was performed using GraphPad Prism 10.

### 2.10 p-IRF3 and p-STAT1 western blot of THP-1 stimulated cells

The THP-1 stimulation assay was performed as previously described (Marr et al., 2010a). THP-1 cells were differentiated using PMA and stimulated with the desired dilution of stimulant (1:10 dilution of heat-killed bacterial suspension in complete RPMI 1640; sterile media for negative “no stimulation” control; 10 μg/mL of *E. coli* K12 LPS) in duplicates. At 4 h post-stimulation, the supernatant was removed, and the THP-1 cells were washed with sterile PBS. The cells were then scraped in 100 μL PBS and stored in Eppendorf tubes at −20°C.

When performing western blot experiments, the cells were denatured and proteins were separated by 12% SDS-PAGE as described before (Fathy Mohamed and Fernandez, 2024). Three such gels were prepared for each stimulation assay. The first gel was stained with PageBlue^™^ protein staining solution (Thermo Scientific). Image Lab Software (RRID:SCR_014210) was used to detect total protein content of the sample lanes. Proteins from the second and third gels were transferred to Immobilon-P polyvinylidene difluoride (PVDF) membranes (Sigma) and immunoblotted for p-IRF3 and p-STAT1, respectively (Fathy Mohamed and Fernandez, 2024). Primary antibodies anti-IRF3 (phosphor S386) antibody EPR2346 (Abcam Cat# ab76493, RRID:AB_1523836) and phospho-Stat1 (Tyr701) (58D6) Rabbit mAb (Cell Signaling Technology Cat# 9167, RRID:AB_561284), as well as, secondary antibody Peroxidase AffiniPure^™^ Goat anti-Rabbit IgG (H + L) (Jackson ImmunoResearch Labs Cat# 111–035-144, RRID:AB_2307391) were used. The proteins were detected using chemiluminescence (ECL^™^ Prime Western Blotting Detection reagent; Cytivia) in the BioRad Imaging System. The membrane was exposed for 360 s, and the image obtained was analyzed using Image Lab Software to calculate band intensities. The band intensities, normalized to the total protein content, were converted as a percentage of *B. parapertussis* WT. One-way ANOVA with Tukey’s multiple comparison test was performed using GraphPad Prism 10.

## 3 Results

### 3.1 Engineering *Bordetella* strains to encode different LPS structures

**Figure 1:**
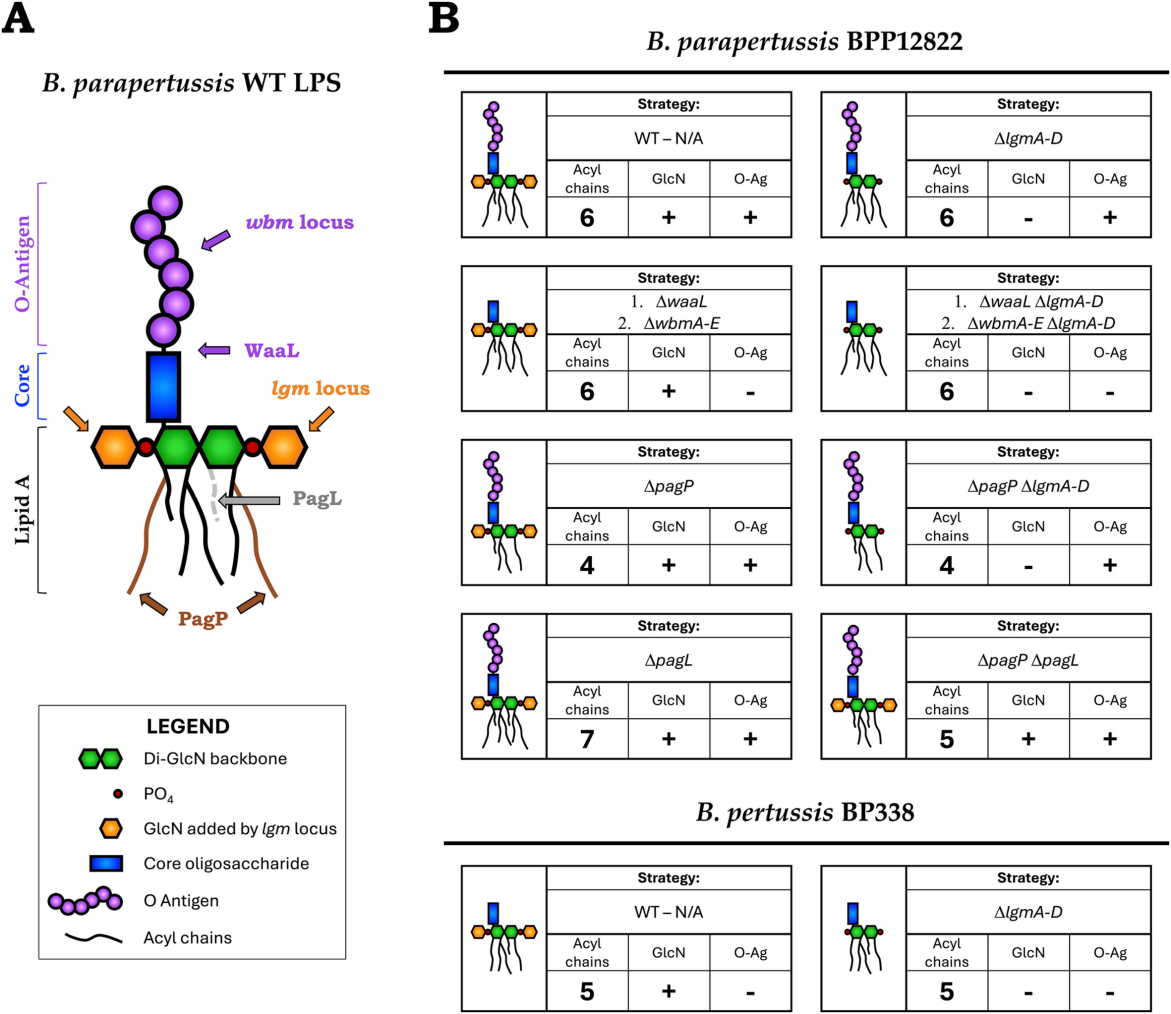
**(A)** Schematic of *B. parapertussis* WT LPS structure with the targets of deleted genes/loci indicated—lipid A of *B. parapertussis* consists of six acyl chains attached to a diglucosamine backbone (green) to which the core (blue) and the O antigen (purple) is attached. Deleting the ligase, WaaL, or the first five genes of the *wbm* locus creates an LPS species devoid of O antigen (purple). The number of acyl chains was modified by deleting PagP, which adds two palmitate groups (brown chains) to lipid A, or PagL, a deacylase that removes an acyl chain (grey dotted). The decoration of the phosphates (red) of the lipid A backbone with glucosamine moieties (orange) was prevented by deleting the *lgm* locus. **(B)** *Bordetella* WT and mutant strains used in the study—each block represents a *Bordetella* strain (WT or mutant), schematic of the encoded LPS structure and the strategy (genes deleted) to obtain said structure. It also indicates the number of acyl chains in the final structure and if it encodes the GlcN modifications and the O antigen.

To further understand how TLR4 differentiates structurally distinct *Bordetella* LPS, *B. pertussis* and *B. parapertussis* strains were genetically engineered to encode LPS with different structural features. First, to investigate if the O antigen biases signaling towards the TRIF pathway, LPS structures with and without the O antigen were engineered. Of the two parent strains, *B. parapertussis* expresses an O antigen due to the presence of an intact *wbm* biosynthesis locus, while, *B. pertussis* lacks an O antigen as the locus was replaced by an insertion sequence (Di Fabio et al., 1992; Preston and Maskell, 2001). The O antigen from *B. parapertussis* was removed by deleting either the ligase (WaaL) that attaches the pre-formed O antigen to the lipid A + core moiety (Mulford and Osborn, 1983; Kalynych et al., 2014) or by deleting the first five genes of the *wbm* locus (*wbmA-E*), which has been shown sufficient to prevent O antigen synthesis (Preston et al., 1999) as indicated in Figure 1A. Thus, hexa-acylated LPS with (*B. parapertussis* WT) and without (*B. parapertussis* Δ*waaL* or Δ*wbmA-E*) an O antigen were generated. Additionally, by altering the number of acyl chains in *B. parapertussis* as outlined below, we generated a penta-acylated LPS expressing an O antigen (*B. parapertussis* Δ*pagP* Δ*pagL*) which could now be compared to the penta-acylated *B. pertussis* WT LPS that lacks the O antigen.

To study the influence of the number of acyl chains on TLR4 signaling, strains encoding lipid A with 4 to 7 acyl chains were created by deleting *pagP* and/or *pagL* from hexa-acylated *B. parapertussis* (El Hamidi et al., 2009). PagP is a palmitoyl transferase that adds a secondary palmitate group to the acyl chains present at the C2 and C3′ positions (Hittle et al., 2015) (Figure 1A). On the other hand, PagL is a lipid A deacylase that removes the 3-hydroxydecanoic acid moiety from the C3 position (Geurtsen et al., 2006). Hence, deleting *pagP*, *pagL* or both together led to LPS species with 4, 7 or 5 acyl chains respectively, that could now be compared to *B. parapertussis* and *B. pertussis* LPS containing 6 and 5 acyl chains, respectively.

Both *B. pertussis* and *B. parapertussis* encode the *lgm* locus responsible for the GlcN modification of the phosphates of the lipid A backbone (Marr et al., 2008; Geurtsen et al., 2009). To study their role in TLR4 signaling in conjunction with other LPS structural features, the entire locus, *lgmA-D*, was deleted from both *B. pertussis* and *B. parapertussis* to create LPS with and without the GlcN modification.

The genes stated above were either deleted singly or in combination, deleting one gene at a time, using a markerless clean deletion protocol. In total, 10 *Bordetella* mutants were created that expressed eight different LPS structures that differed in the presence or absence of the O antigen, the number of acyl chains, and/or the presence or absence of the GlcN modification as indicated in Figure 1B. All *Bordetella* mutants were complemented with the respective deleted genes, either on an aTC inducible pIG10 plasmid (for *waaL*, *wbmA-E*, *pagP* and *pagL*) or the mini-Tn7 transposase system (for *lgmA-D*) (Choi et al., 2008). These strains, their respective WT strain as well as *E. coli* K12 LPS were used in subsequent experiments to study TLR4 recognition and signaling.

### 3.2 Tricine-SDS-PAGE and MALDI-TOF analysis validate the LPS structure of mutants

**Figure 2:**
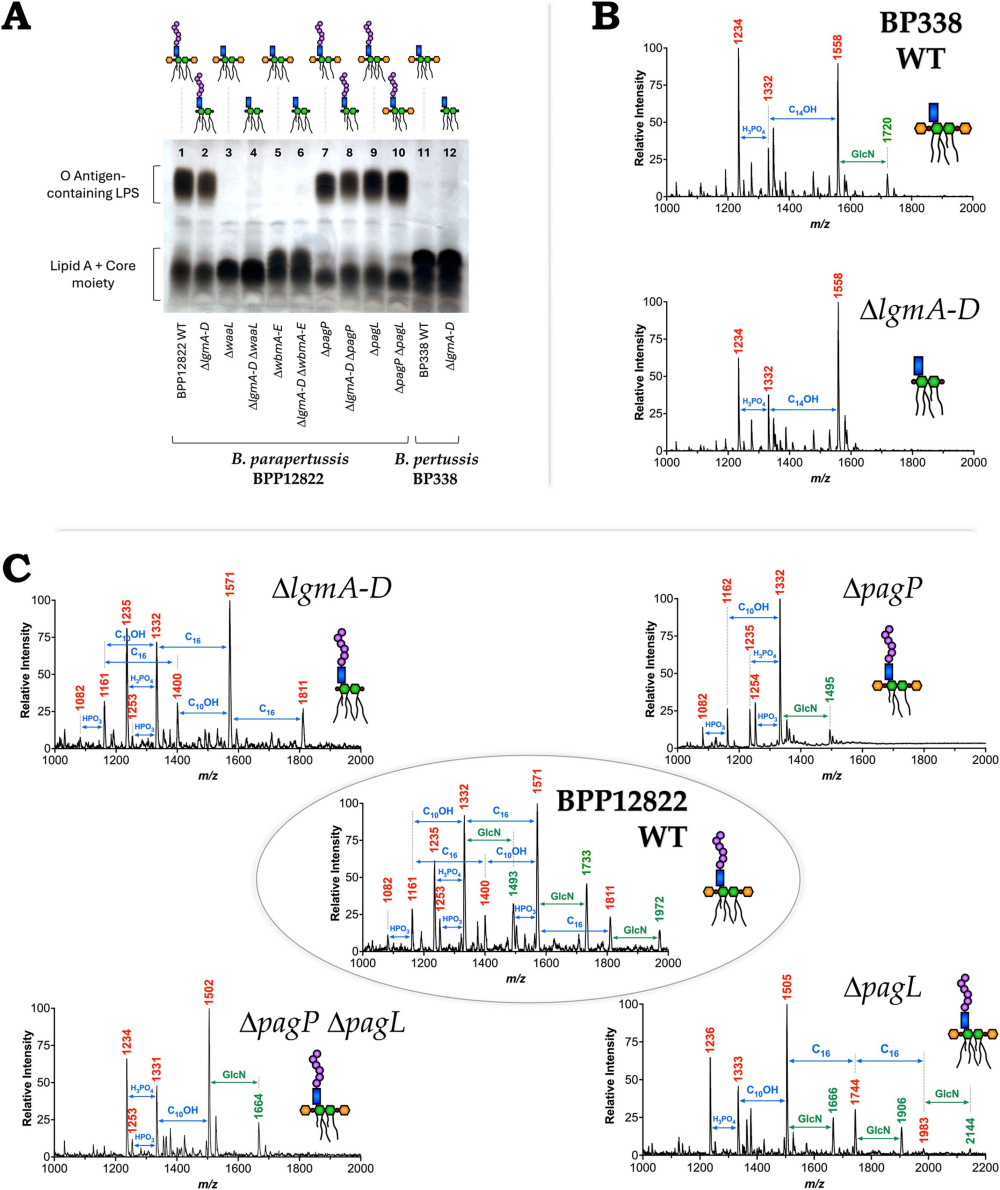
Validation of the structures of *Bordetella* LPS mutants. **(A)** Tricine-SDS-PAGE analysis of the variants—DNase I, RNase and proteinase K treated lysates were subjected to tricine-SDS-PAGE and stained using silver staining. **(B)** Negative-ion MALDI-TOF mass spectra of lipid A isolated from *B. pertussis* strains—WT (top) and Δ*lgmA-D* mutant (bottom). **(C)** Structural analysis of lipid A of select *B. parapertussis* variants—WT (center) and going clockwise: Δ*lgmA-D* mutant (top left), Δ*pagP* mutant (top right), Δ*pagL* mutant (bottom right) and Δ*pagP* Δ*pagL* mutant (bottom left). Predicted LPS structure is indicated beside each strain.

After confirming the deletion of the respective genes in the mutants using PCR, evidence for the presence or absence of the O antigen in the engineered LPS was obtained using tricine-SDS-PAGE and visualized using silver-staining. Bands corresponding to the lipid A + core moiety (bottom band) and the O antigen-containing LPS (top smear) were noted (Figure 2A). The homopolymeric O antigen-containing LPS of *B. parapertussis* WT is seen as a smear at the top of the gel (Lane 1). The mutants lacking the O antigen (Δ*waaL*, Δ*wbmA-E*, Δ*lgmA-D* Δ*waaL* and Δ*lgmA-D* Δ*wbmA-E*; Lanes 3 to 6) lack the smear at the top, confirming the loss of O antigen. All other mutants containing modifications to the lipid A alone show no change in the expression of the O antigen as expected (Lanes 2 and 7 to 10). *B. pertussis* WT and Δ*lgmA-D* mutants naturally do not express the O antigen as confirmed by the lack of the O antigen-containing LPS smear (Lanes 11 and 12). However, the lipid A + core band of *B. pertussis* migrates slower than that of *B. parapertussis*, presumably due to the addition of the distal trisaccharide unit to the core by the *wlb* locus, which is absent in *B. parapertussis* (Allen et al., 1998). The *B. parapertussis* O antigen mutants (Δ*waaL*, Δ*wbmA-E*, Δ*lgmA-D* Δ*waaL* and Δ*lgmA-D* Δ*wbmA-E*), when complemented with the respective deleted genes, demonstrate successful complementation of *waaL* or *wbmA-E* as indicated by the reappearance of the O antigen-containing LPS smear (Supplementary Figure S1).

Evidence for the absence of the GlcN moiety (in the Δ*lgmA-D* mutants) and of the number of acyl chains present (in the Δ*pagP* and Δ*pagL* mutants) was acquired via MALDI-TOF. Lipid A was extracted using mild acid hydrolysis and analyzed using negative, linear ion-mode MALDI-TOF. While MALDI-TOF was performed for all mutants, Figures 2B,C illustrate the spectra of select mutants. Peaks of interest are labeled in red or green. Other *m*/*z* in the spectra correspond to micro-heterogeneity due to changes in hydroxylation and acyl chain length. The top panel of Figure 2B shows the spectra of *B. pertussis* WT LPS. The peak at *m*/*z* 1,558 represents the penta-acylated LPS species, followed by tetra-acylated species at *m*/*z* 1,332 and 1,234. The addition of a single GlcN moiety is observed as an addition of *m*/*z* 161 resulting in a peak at *m*/*z* 1,720 (green) (Marr et al., 2010a). The Δ*lgmA-D* mutant lacks this peak confirming the loss of this moiety (Figure 2B lower panel).

The central panel of Figure 2C shows the spectra of *B. parapertussis* WT LPS. Similar to what was previously reported for *B. parapertussis* (El Hamidi et al., 2009; Hittle et al., 2015), our data shows peaks at *m*/*z* 1,332, 1,571, and 1,811 that correspond to LPS species with 4, 5 and 6 acyl chains, respectively. The corresponding GlcN modified species, with *m*/*z* 161 higher, are highlighted in green (*m*/*z* 1,493, 1,733 and 1,972 respectively). The peaks corresponding to the GlcN modification are absent in the Δ*lgmA-D* mutant (top left). The Δ*waaL* and Δ*wbmA-E* mutants, which did not have any modifications to their lipid A structure, had the same mass spectra as *B. parapertussis* WT (Supplementary Figure S2). Likewise, the Δ*lgmA-D* Δ*waaL* and Δ*lgmA-D* Δ*wbmA-E* had mass spectra similar to the Δ*lgmA-D* mutant (Supplementary Figure S2). PagP adds two palmitate groups to *B. parapertussis* LPS, with *m*/*z* 238.4 each. Correspondingly, the Δ*pagP* mutant (top right) lacks the peaks at *m*/*z* 1,571 and 1,811 corresponding to the penta-and hexa-acylated species. Deletion of PagL prevents the deacylation of a C_10_-OH group with *m*/*z* 170.25. Hence, the Δ*pagL* mutant (bottom right) is seen to have an additional peak at *m*/*z* 1,983 indicative of a hepta-acylated species, followed by the addition of a single GlcN at *m*/*z* 2,144. Also, the Δ*pagP* Δ*pagL* double mutant (bottom left) corresponded to the loss of two palmitate groups at *m*/*z* 238.4 each and the addition of C_10_-OH group at *m*/*z* 170.25, resulting in a final loss of *m*/*z* 306.55. Thus, the double mutant has a major peak corresponding to a penta-acylated species with *m*/*z* 1,502, that is *m*/*z* 309 less than the hexa-acylated WT LPS at *m*/*z* 1,811.

### 3.3 Loss of GlcN modification and under-acylation of lipid A significantly reduce TLR4-mediated NFκB activation while the O antigen and the seventh acyl chain do not have any impact

**Figure 3:**
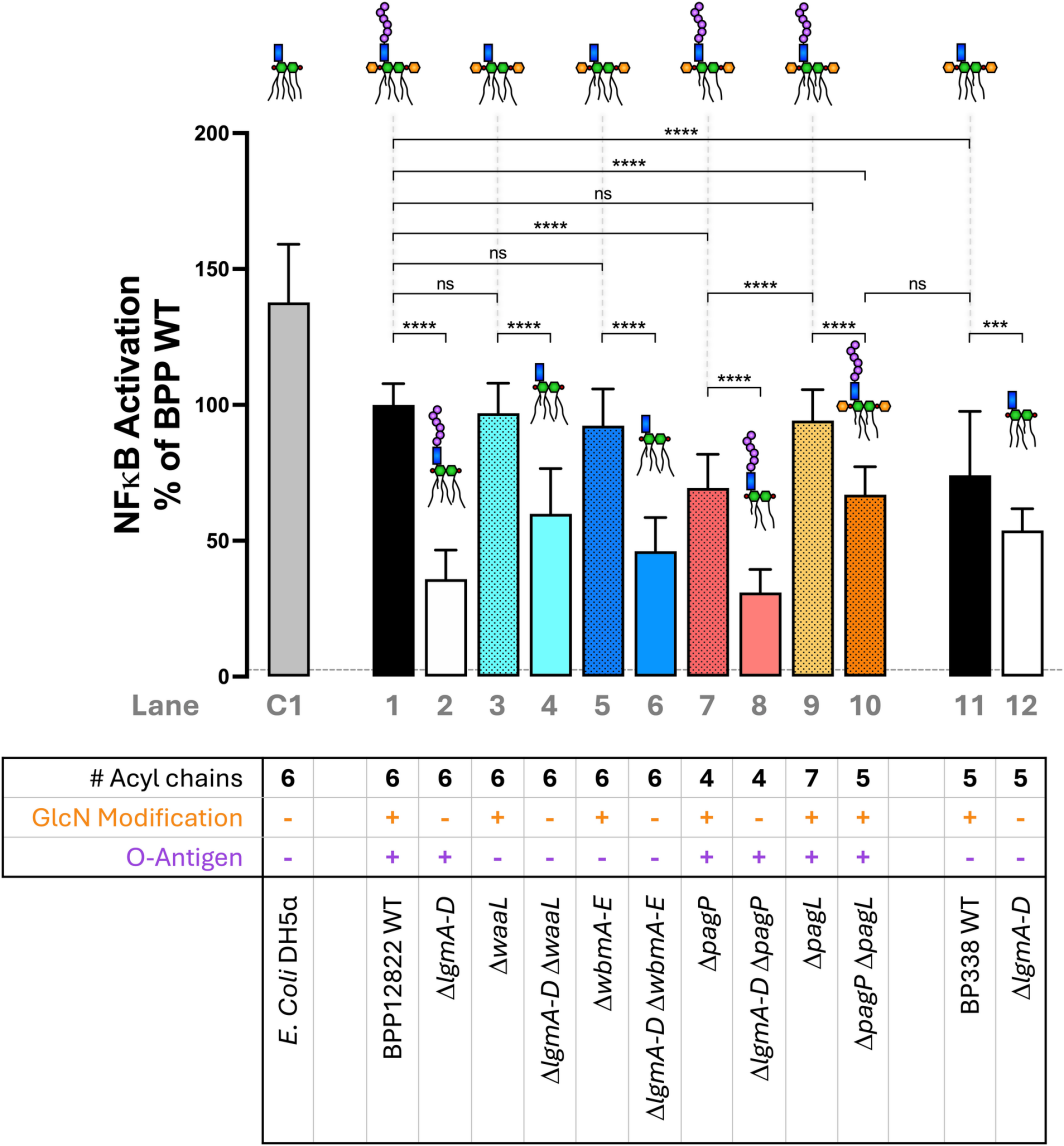
Absence of GlcN modification and under-acylation of lipid A, but not the loss of the O antigen nor over-acylation of lipid A, significantly reduce NFκB activation via TLR4/MD-2—A 1:100 dilution of heat-killed bacteria was used to stimulate HEK-Blue^™^ hTLR4 reporter cells to measure NFκB activation 15 min post-mixing with the QUANTI-Blue reagent. The assay was repeated 5 times in total with 5 technical replicates each. The absolute absorbance readings were converted as a percentage of *B. parapertussis* WT and plotted. The histograms show the mean + standard deviation. One-way ANOVA with Tukey’s multiple comparison test was performed using GraphPad Prism 10. ns, not significant; ^***^p < 0.001 and ^****^p < 0.0001. Predicted LPS structure and lane number are indicated beside each strain. Dotted line represents average Null2 readings across samples.

Next, the ability of the engineered *Bordetella* LPS to activate TLR4-mediated NFκB signaling was investigated. Heat-killed bacteria (at 1:100 dilution) from each strain were introduced to an NFκB reporter cell line, HEK-Blue^™^ hTLR4 cells, that expresses human TLR4/MD-2 and CD14. HEK-Blue^™^ Null2 was used as a control to rule out NFκB activation by endogenously expressed pattern recognition receptors. The degree of NFκB activation was measured 15 min post-mixing with the QUANTI-Blue reagent as an absorbance readout at 650 nm (Figure 3). Hexa-acylated *E. coli* DH5α was used as a control (Lane C1). *B. parapertussis* WT (Lane 1), with a hexa-acylated lipid A, did not induce as much NFκB as *E. coli* LPS (Lane C1), but it activated NFκB stronger than the penta-acylated *B. pertussis* LPS (Lane 11). The most prominent phenotype observed across the board was the significant reduction in NFκB activation upon the deletion of the *lgm* locus when compared to the WT or their respective single mutant parent in the case of a double mutant (Lanes 2, 4, 6, 8 and 12). Furthermore, the reduction in NFκB activation on deleting the *lgm* locus in *B. parapertussis* WT is much more striking than that observed in *B. pertussis* (Lanes 1 and 2 vs. Lanes 11 and 12).

Secondly, the O antigen had no impact on NFκB activation. *B. parapertussis* LPS lacking O antigen either by the deletion of the ligase (Δ*waaL*; Lane 3) or the biosynthesis locus (Δ*wbmA-E*; Lane 5) showed equivalent NFκB activation as the WT strain (Lane 1). Also, penta-acylated *Bordetella* LPS that expresses the O antigen (*B. parapertussis* Δ*pagP* Δ*pagL*; Lane 10) and one that does not (*B. pertussis* WT; Lane 11) activated NFκB to equal degrees. Lastly, the number of acyl chains had a nuanced impact on NFκB signaling. Having an extra acyl chain (Δ*pagL*; Lane 9) compared to the *B. parapertussis* WT (Lane 1) was neither beneficial nor detrimental to NFκB activation. However, reducing the number of acyl chains (to 4 in Δ*pagP* mutant or 5 in Δ*pagP* Δ*pagL* double mutant; Lanes 7 and 10) reduced NFκB activation. Any reduction of NFκB activation was restored to WT levels upon complementation of the respective mutants (Supplementary Figure S3).

### 3.4 *Bordetella* O antigen facilitates TLR4-mediated TRIF pathway activation

TLR4-mediated NFκB activation (as measured above) is triggered by both the MyD88 and the TRIF pathways. Of these, the MyD88 pathway is key for the immediate and strong activation of NFκB, while the TRIF pathway, though majorly involved in activating IRF3 and Type I interferons, triggers the late-phase activation of NFκB (Lu et al., 2008). Hence, we investigated if these structural changes in LPS biased the TLR4-mediated signaling toward the TRIF pathway.

To assess TRIF-mediated IRF3 activation, two techniques were employed. First, heat-killed bacteria were used to stimulate THP-1-Dual cells, which consist of a Lucia luciferase reporter under the control of the interferon-stimulated response element (IRSE) which is induced by phosphorylated IRF3 or Type 1 interferon-mediated STAT signaling. Luciferase activity was then measured at 24 h using the flash detection method and expressed as relative light units. *E. coli* K12 LPS was used as a positive control.

**Figure 4:**
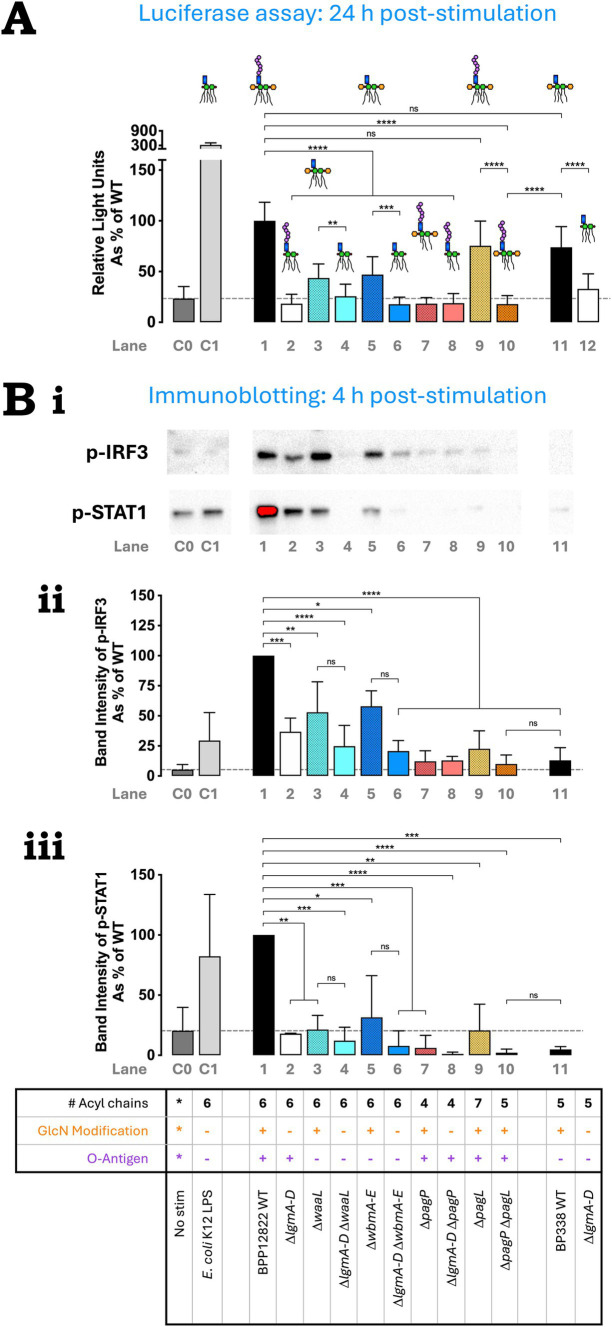
O antigen is important for TLR4-mediated TRIF pathway activation. **(A)** IRF3 activation by *B. parapertussis* strains using THP-1 Dual reporter assay—heat-killed bacteria were used to stimulate reporter THP-1 Dual^™^ cells for 24 h and the extent of IRF3 stimulation was measured as relative light units. The assay was repeated 4 times in total with 4 technical replicates each. The absolute luminescence readings were converted as a percentage of *B. parapertussis* WT readings and plotted. The histograms show the mean + standard deviation. Mixed-effects analysis with Tukey’s multiple comparison test was performed using GraphPad Prism 10. **(B)** p-IRF3 and p-STAT1 western blot indicate reduction in IRF3 and STAT1 phosphorylation upon the deletion of the *lgm* locus, O antigen and/or altering the number of acyl chains—heat-killed bacteria were used to stimulate PMA-differentiated THP-1 cells. Cells were collected at 4 h, lysed and immunoblotted for p-IRF3 and p-STAT1. **(Bi)** p-IRF3 and p-STAT1 western blot—a representative image of three repeats for the immunoblotting of p-IRF3 and p-STAT1 is shown. Quantification of p-IRF3 **(Bii)** and p-STAT-1 **(Biii)**—the band intensity for each repeat was calculated using Image Lab software, normalized to the total protein content and expressed as a percentage of *B. parapertussis* WT. Histograms show the mean + standard deviation. One-way ANOVA with Tukey’s multiple comparison test was performed using GraphPad Prism 10. ns, not significant; ^*^p < 0.05, ^**^, *p* < 0.01, ^***^p < 0.001, and ^****^p < 0.0001. Predicted LPS structure and lane numbers are indicated beside each *Bordetella* strain. Dotted line represents mean “no stimulation” reading.

*B. parapertussis* WT showed high levels of IRF3 activation which was comparable to *B. pertussis* WT (Figure 4A; Lanes 1, 11). Both O antigen mutants (Δ*waaL* and Δ*wbmA-E*; Lanes 3 and 5) showed a significant reduction in IRF3 activation. Altering the number of acyl chains down to 4 or 5 also abolished IRF3 activation (Lanes 7, 10), while increasing it to 7 had no impact (Lane 9). Intriguingly, the penta-acylated *B. parapertussis* Δ*pagP* Δ*pagL* mutant (Lane 10) activated IRF3 to a significantly lower extent than *B. pertussis* WT (Lane 11) despite it having an O antigen (whose presence seemed to increase IRF3 activation in *B. parapertussis* WT compared to its O antigen mutants). Upon the absence of the GlcN modification in respective single or double mutants, negligible IRF3 activation was observed (Lanes 2, 4, 6, 8, 12). Like that observed in NFκB activation, the drop in IRF3 activation in the *B. parapertussis* Δ*lgmA-D* mutant when compared to its WT is more dramatic compared to that observed in *B. pertussis* (Lanes 1 and 2 vs. Lanes 11 and 12).

The above assay was not sufficiently sensitive to detect immediate IRF3 activation at 4 h post-stimulation. Thus, western blot was used to detect the presence of phosphorylated IRF3 (p-IRF3) and phosphorylated STAT1 (p-STAT1) 4 h after the stimulation of PMA-differentiated THP-1 cells with heat-killed bacteria. A representative western blot image for p-IRF3 and p-STAT1 is shown in Figure 4B,i. The band intensity of p-IRF3 and p-STAT1 was quantified using Image Lab software and represented as a percentage of *B. parapertussis* WT in Figures 4B,ii,iii respectively. Except for *B. parapertussis* Δ*pagL* mutant (Lane 9) and *B. pertussis* WT (Lane 11), the western blot data at 4 h replicated the luciferase reporter assay results observed at 24 h. *B. parapertussis* had the highest intensity of p-IRF3 and p-STAT1 bands (Figures 4B,ii,iii; Lane 1). Deleting the O antigen caused a moderate reduction in p-IRF3 band intensity (Lanes 3, 5), while deleting the *lgm* locus (Lanes 2, 4, 6, 8, 12) or altering the number of acyl chains (Lanes 7, 9, 10) caused a significant reduction (Figure 4B,ii). Contrary to the luciferase assay, the hepta-acylated Δ*pagL* mutant (Lane 9) had significantly less p-IRF3 band intensity than the WT (Lane 1) at 4 h post-stimulation. Similarly, *B. pertussis* also had minimal IRF3 activation (Figure 4B,ii; Lane 11). Additionally, any structural changes to *B. parapertussis* WT LPS led to significantly lower STAT1 activation in all strains when compared to the WT (Figure 4B,iii; Lane 1). *B. pertussis* also had minimal STAT1 activation at 4 h (Figure 4B,iii; Lane 11).

## 4 Discussion

Overall, all three LPS features studied: the GlcN modification of the backbone phosphates, the O antigen and the number of acyl chains, were observed to alter TLR4-mediated signaling, albeit in their own unique ways.

Previous studies on TLR4 signaling by *B. pertussis* LPS lacking GlcN modification showed a reduction in THP-1 macrophage-mediated cytokine production downstream of both the MyD88 pathway (e.g., IL-6, TNFα) and the TRIF pathway (e.g., IP-10, MCP-1, RANTES) (Marr et al., 2010a). Upon further investigation, a group of four negatively charged amino acid residues on human TLR4 were collectively shown to be important for its interaction with the positively charged GlcN moiety of *B. pertussis* LPS, thereby facilitating dimerization and subsequent NFκB activation (Maeshima et al., 2015). In this study, we were not only able to replicate the impact of GlcN modification on TLR4 signaling in *B. pertussis* but in hexa-acylated *B. parapertussis* as well. The lack of this modification was found to consistently and significantly reduce both NFκB and IRF3 activation in both strains. Additionally, deleting the *lgm* locus in *waaL*, *wbmA-E* or *pagP* mutants further reduced NFκB and IRF3 activity. Hence, this study provides compelling evidence that the GlcN modification had an overarching dominant influence on both TLR4-mediated signaling pathways, overriding the effects of the O antigen’s presence or absence and the number of acyl chains attached to lipid A. Furthermore, similar to trends reported by Geurtsen et al. (2009), whereby an insertional inactivation of *arnT* (i.e., *lgmB*) in *B. parapertussis* led to a greater drop in IL-6 production when compared to that in *B. pertussis*, we also observed a greater drop in both NFκB and IRF3 activation upon the deletion of the *lgm* locus in *B. parapertussis* compared to *B. pertussis*. These observations underscore the importance of the role of the GlcN moiety in the initial interaction and dimerization of TLR4/MD-2—LPS complexes which thereby dictates the overall activation of TLR4-mediated signaling, impacting the MyD88 and the TRIF pathway equally. While the presence of the GlcN moiety in the WT strains increases the visibility of the bacteria to the human immune system through TLR4, it has been proven beneficial to the bacteria by increasing resistance to cationic antimicrobial peptides and contributing to the integrity of the outer membrane (Shah et al., 2014). However, the benefits of the GlcN modification in *B. parapertussis* are unclear.

Secondary to the GlcN modification, the structural feature influencing TLR4 signaling the most was the number of acyl chains attached to lipid A. Hexa-acylated *E. coli* and *B. parapertussis* strongly activated NFκB and IRF3. In contrast, under-acylation to 4 or 5 acyl chains (in *B. parapertussis* Δ*pagP* and Δ*pagP* Δ*pagL* respectively) moderately reduced NFκB activation and completely abrogated signaling via the TRIF pathway. A study examining *E. coli* LPS and TLR4/MD-2 interaction showed that five of the acyl chains of LPS fit into the MD-2 pocket, while the sixth lay exposed and free to facilitate dimerization by interacting with hydrophobic residues on the second TLR4 (Park et al., 2009). Consequently, it stands to reason that under-acylation would change the fit of the LPS in the MD-2 pocket or prevent the exposure of an acyl chain and thereby weaken TLR4 dimerization and consequently, downstream signaling. This reasoning supports the results observed in our study as well as those seen with other under-acylated LPS like Lipid IVA, *R. sphaeroides* and LPS1435/1449 variant of *P. gingivalis* (Meng et al., 2010; Herath et al., 2013; Anwar et al., 2015). Thus, we deduce that under-acylation of *B. parapertussis* LPS could weaken TLR4 dimerization to an extent where it moderately signals via the cell surface-MyD88 pathway but hinders the endocytosis of the dimer and/or the activation of the TRIF pathway. On the other hand, increasing the number of acyl chains to 7 (*B. parapertussis* Δ*pagL*) did not impact both pathways at 24 h indicating that the extra acyl chain, presumably also exposed from the MD-2 pocket, does not hinder nor benefit TLR4-mediated signaling over time. However, it remains unclear why hepta-acylation affected early (4 h) TRIF pathway activation alone, unless explained by differences in experimental protocol.

Last of all, the presence or absence of the O antigen in either penta-or hexa-acylated *Bordetella* species did not alter NFκB activation. However, the loss of the O antigen in *B. parapertussis* led to a significant reduction in TRIF pathway activation at both 4 h and 24 h. This work corroborates studies by Fedele et al. (2008) and Zanoni et al. (2012) who showed that LPS with an O antigen induced superior DC maturation and IFN-β response respectively, when compared to its O antigen lacking LPS species, presumably due to its interaction with CD14. These studies, along with ours, support the theory that the O antigen interacts with the TLR4 cofactor, CD14, which is indispensable for TRIF pathway activation. This interaction thus promotes the endocytosis of the TLR4/MD-2—LPS dimer, biasing signaling towards the TRIF pathway without affecting the MyD88 pathway at the cell surface (Gangloff et al., 2005; Jiang et al., 2005; Zanoni et al., 2011; Zanoni et al., 2012). On the contrary, the penta-acylated species with and without the O antigen (*B. parapertussis* Δ*pagP* Δ*pagL* vs. *B. pertussis* WT) behaved differently. At 4 h post THP-1 stimulation, both species induced equally negligible levels of p-IRF3 and p-STAT1 despite one expressing the O antigen and the other not. Additionally, at 24 h post-stimulation, *B. pertussis* WT (without the O antigen) activated IRF3 significantly more than *B. parapertussis* Δ*pagP* Δ*pagL* expressing the O antigen, contrary to that observed in hexa-acylated LPS species. Inherent differences in LPS structure between *B. pertussis* and *B. parapertussis* such as acyl chain length and position, or the presence of the distal trisaccharide may contribute to the conflicting trends observed in IRF3 activation. Also, the influence of differences in experimental protocol, length of stimulation, or antigens encoded by *B. pertussis* and *B. parapertussis* on the observed results cannot be ruled out.

This study has given us a much deeper insight into how the GlcN modification, the number of acyl chains and the O antigen of *Bordetella* LPS influence the activation of TLR4-mediated MyD88 and TRIF pathways. In summary, the GlcN modification had an overarching effect over the O antigen and lipid A acylation, with its absence strongly reducing both MyD88 and TRIF pathway activation. Next, the under-acylation of LPS (to 4 or 5 acyl chains) partially reduced NFκB activation and abolished TRIF pathway activation while hexa-and hepta-acylated LPS equally and strongly activated NFκB and IRF3. Lastly, while not impacting the MyD88 pathway, the *Bordetella* O antigen biased signaling towards the TRIF pathway. This knowledge is not only helpful in understanding the interaction between LPS and TLR4 and the factors influencing downstream signaling, but also aids in creating engineered LPS species that can specifically modulate the immune response generated. Bacterial LPS structure can be tailored to delicately tune the MyD88 vs. TRIF response to enhance the generated immune response and memory, thereby informing vaccine design.

## Data Availability

All data for this study are provided within the manuscript and supplementary information files, further inquiries can be directed to the corresponding author.

## Footnotes

^1^Centers for Disease Control and Prevention (CDC).

^2^European Centre for Disease Prevention and Control (ECDC).

^3^Public Health Agency of Canada (PHAC).
