# Supplementary material for: Determining the Bordetella LPS structural features that influence TLR4 downstream signaling
Source: Front Microbiol. 2025 Feb 25;16:1540534. doi: 10.3389/fmicb.2025.1540534 (PMC11895810; doi:10.3389/fmicb.2025.1540534)
Supplement: Supplementary file 1 [file Data_Sheet_1.docx]

Supplementary Material

# Supplementary Figures

## Supplementary Figures

**Supplementary Figure 1:** Tricine-SDS-PAGE of lysates from *B. parapertussis* O antigen mutants complemented with *waaL* or *wbmA-E* respectively have the O antigen-containing LPS smear which was absent in the mutants.

**Supplementary Figure 2:** **MALDI-TOF mass spectra of lipid A isolated from O antigen mutants –** O antigen deletion mutants, Δ*waaL* and Δ*wbmA-E*, exhibited similar mass spectra as *B. parapertussis* WT LPS. Δ*lgmA-D* Δ*waaL* and Δ*lgmA-D* Δ*wbmA-E* double mutants showed mass spectra identical to the Δ*lgmA-D* mutant.

**Supplementary Figure 3:** **Complementation of all deleted genes in the *B. parapertussis* and *B. pertussis* mutants restores NFκB activation to WT levels –** 1 in 1000 dilution of heat-killed bacteria were used to stimulate HEK-Blue^TM^ hTLR4 reporter cells, with NFκB activation measured 30 min post-mixing with the Quanti-Blue reagent. The absolute absorbance readings were converted as a percentage of *B. parapertussis* WT. The graph represents data compiled from 4 assays, with 5 technical replicates each, conducted on multiple days. Error bars indicate the standard deviation.
